# Avian Pathogenic *Escherichia coli*: An Overview of Infection Biology, Antimicrobial Resistance and Vaccination

**DOI:** 10.3390/antibiotics13090809

**Published:** 2024-08-26

**Authors:** Amyleigh Watts, Paul Wigley

**Affiliations:** 1 Institute of Infection, Veterinary and Ecological Sciences, University of Liverpool, Leahurst Campus, Neston CH64 7TE, UK; amyleigh.watts@liverpool.ac.uk; 2 Bristol Veterinary School, University of Bristol, Bristol BS40 5DU, UK

**Keywords:** APEC, chicken, colibacillosis, ESBL, fluoroquinolone, colistin, vaccine, probiotic, bacteriophage, antimicrobial stewardship

## Abstract

Avian Pathogenic *Escherichia coli* (APEC) is an extraintestinal pathotype of *E. coli* that leads to a range of clinical manifestations, including respiratory, systemic and reproductive infections of chickens in both egg and meat production. Unlike most *E. coli* pathotypes, APEC is not defined by specific virulence genes but rather is a collection of several distinct genotypes that can act as both primary and secondary pathogens leading to colibacillosis. Recent measures to reduce antimicrobials both as growth promoters and as flock-level therapeutics are considered to have led to increased numbers of animals affected. Nevertheless, antimicrobial resistance is a considerable problem in APEC, with resistance to third and fourth-generation cephalosporins via extended-spectrum beta-lactamases (ESBLs), fluoroquinolones and colistin seen as a particular concern. The need to control APEC without antimicrobial use at the flock level has seen an increased focus on vaccination. Currently, a few commercial vaccines are already available, and a range of approaches are being applied to develop new vaccines, and other controls, such as bacteriophage or probiotics, are attracting interest. The lack of a single defined APEC genotype presents challenges to these approaches.

## 1. Introduction

Avian Pathogenic *Escherichia coli* (APEC), along with the human pathogenic neonatal meningitis-associated *E. coli* (NMEC) and Uropathogenic *E. coli* (UPEC), APEC is classified as an extraintestinal pathogenic *E. coli* (ExPEC). ExPEC are pathotypes distinct in their genotypes and virulence genes to those associated with diarrheal disease or commensal *E. coli* within the gut. APEC is a major pathogen of poultry production affecting fast-growing broiler chickens (meat-producing), laying hens (egg-producing) and breeding chickens, along with turkeys. APEC can frequently act as a secondary or opportunistic pathogen following infection with respiratory viruses or a consequence of poor management, but it also has the capacity to be a primary pathogen in healthy flocks. Furthermore, *E. coli* associated with chicken production are seen as a major reservoir of antimicrobial-resistant bacteria. The control of APEC is likely to require a range of approaches, but given its apparent increase in prevalence with decreased use of antimicrobials and further pressures to decrease their use, better vaccines or other controls are needed [1].

## 2. Infection Biology

**Figure 1:**
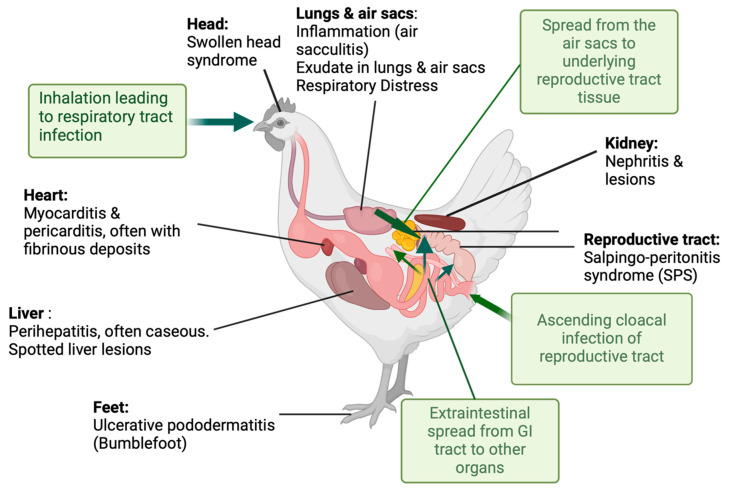
Sites and clinical manifestations of APEC infections along with main routes of transmission into and within infected birds (in shaded boxes) Made with www.BioRender.com.

Although the exact route of extraintestinal *E. coli* infection in the chicken remains unclear, translocation from the intestinal tract or entry via inhalation is considered the most likely route. However, in egg peritonitis, ascending infection via the oviduct has been proposed. The absence of a diaphragm in birds means the intestines closely abut the air sacs of the respiratory system, meaning transfer from the gut is a distinct possibility. Colibacillosis is a disseminated APEC infection affecting birds of all ages and can lead to high mortality and morbidity, reduced productivity and rejection of carcasses at slaughter. Common presentations of colibacillosis include air sacculitis, cellulitis, peritonitis, salpingitis, swollen head syndrome, omphalitis, and pericarditis [1]. Infection with pathogenic *E. coli* can occur following inhalation of infected dust particles or by consumption of food or water contaminated with faeces and subsequent extraintestinal translocation [2]. In laying hens bred for intensive egg production, the cloaca, the common opening of the gastrointestinal, urinary and reproductive tracts, can also be an entry point for APEC, leading to salpingo-peritonitis syndrome (SPS), sometimes called egg peritonitis. In hens with SPS, vertical transmission can occur during egg formation [3]. APEC can also be transmitted via faecal contamination of the egg surface, allowing it to penetrate the membrane, causing yolk sac infection and subsequent infection in ovo or in newly hatched chicks [2]. Figure 1 illustrates the potential entry routes of APEC and organs affected by infection.

Despite its importance to the poultry industry, APEC cannot be defined as a single pathotype. In terms of serotypes, O1, O2 and O78 are most frequently associated with APEC [3], with O145, a serotype most usually associated with cattle and Shiga-like toxin-producing *E. coli* (STEC), emerging as a problem in China. More recently, whole genome sequencing approaches have shown the O78 serotype consists of two distinct genomic lineages, ST-23 in phylogroup C and ST-117 in phylogroup G [4]. The O1 and O2 serotypes belong to a third lineage comprising three sub-populations in phylogroup B2: ST-95, ST-140 and ST-428/ST-429. These are likely to form the majority of ‘true’ extraintestinal APEC capable of primary infection. However, the matter is complicated by the fact that many gut-associated ‘commensal’ strains can act as opportunistic pathogens in immunosuppressed, stressed or virally infected birds. Though many virulence genes are associated with APEC, as yet no single gene or pathogenicity island has been identified as being exclusively present in APEC [3,5]. Whilst virulence-associated genes (VAGs) are often used to define APEC, this remains, at best, an imprecise tool. The virulence factors themselves consist of a range of adhesins such as fimbriae, haemolysins, toxins and siderophores, along with genes associated with serum survival and crossing the blood–brain barrier; these have been extensively reviewed previously [6,7]. For example, a longitudinal analysis of systemic *E. coli* isolated from dead birds on broiler farms showed 17.90% of *E. coli* isolates carried no VAGs. Amongst those which did, there was high profile diversity with no apparent VAG profile linked entirely with disease [8,9]. These findings demonstrate the opportunistic nature of *E. coli*, which usually exists as a commensal organism in the gastrointestinal tract. Certainly, a host can be predisposed to disease by factors such as stress, caused by high stocking densities and poor animal husbandry, prior infection (e.g., commonly after infectious bronchitis virus and Newcastle Disease), or concurrent infection with an immunosuppressive disease such as infectious bursal disease [2]. In a similar survey of reproductive tract infection, Collingwood et al. reported that whilst there was still high variation, four VAGs were more commonly associated with disease: *iss* (associated with serum resistance), *iucC* and *iroN* (iron acquisition), and *hlyF* (hemolysin) [5].

## 3. APEC as a Potential Zoonosis

APEC has been considered to have zoonotic potential due to its close genetic relationship to human ExPEC—particularly Uropathogenic *E. coli* (UPEC)—and Neonatal Meningitis *E. coli* (NMEC), and that foodborne transmission occurs in several enteric-associated pathotypes [10], although a firm association has not been established. Several APEC strains have been shown to be capable of causing similar diseases to human-associated ExPEC, including in the rat and mouse meningitis models [11,12]. Conversely, UPEC is capable of producing infection in the chicken reproductive tract in experimental challenge [13].

APEC’s potential to be transmitted in the food chain is perhaps of more concern. Drug-resistant APECs can enter the food chain and be isolated from retail chicken [14,15], and there is considerable pathogenic potential in emerging APECs such as ST117 [16]. However, it should be considered that foodborne and waterborne pathogenic *E. coli* such as STEC, Enterotoxigenic *E. coli* (ETEC) and Enteroaggregative *E. coli* (EAEC) are more adapted to colonise the gastrointestinal tract, and many produce toxins that may act on the gut epithelium or endothelial cells to cause disease.

## 4. Use of Antimicrobials and AMR in APEC

Antimicrobial drugs, usually administered at the flock level, have been employed extensively to control APEC both therapeutically and to an extent as a prophylactic measure. Unsurprisingly, this ‘blanket’ use of treating the whole flock rather than individually infected birds has led to extensive resistance [17]. Defining resistant APEC versus general resistance in *E. coli* associated with poultry encounters the problem of needing a more precise definition of APEC. In the *E. coli* population as a whole, the problem of resistance is well-defined globally with resistance to critically important antimicrobials a particular concern [18]. The use of antimicrobials as growth promoters in chicken production has long been known to be a risk of driving resistance and was recognised more than 50 years ago by the Swann report in the UK [19]. This led to a prohibition on the use of therapeutic drugs as growth promoters and, in turn, a complete ban on antimicrobial growth promoters within the European Union (EU), although many countries have been slow to follow this lead [20]. Recent evidence has shown that treatment of broiler chickens with bacitracin, a common growth promoter, and enrofloxacin, a commonly used therapeutic in poultry, leads to selection for resistant strains of Enterobacteriaceae and that the use of enrofloxacin, in particular, leads to selection for multi-drug-resistant *E. coli* [21]. The problem of excessive antimicrobial use in the poultry industry has not gone unnoticed or been ignored. In the UK, voluntary stewardship of responsible use has reduced the use of antimicrobials by more than 75% in the broiler industry over the last decade, with no use of third and fourth-generation cephalosporins or colistin and very limited use of fluoroquinolones recorded [22,23]. Within the EU, the use of prophylactic and flock-level treatment with antimicrobials was banned in 2022, with only treatment of individual animals allowed. The long-term effects of these approaches are yet to be seen in terms of resistance [20].

Those studies on AMR, specifically in APEC, have tended to centre upon diseased or dead birds in outbreaks of colibacillosis. Levels of resistance are higher in APEC than in a range of other bacterial avian pathogens, with a global review indicating widespread resistance to fluoroquinolones and the presence of ESBLs in APEC from several countries, perhaps reflecting both the ubiquity of APEC and the extensive use of antimicrobials against infection [24]. Typically, high levels of resistance to older and widely used drugs such as ampicillin and tetracyclines were found in around 90% of isolates. Of specific concern are resistance to fluoroquinolones, colistin and the presence of ESBLs in APEC and their potential to enter the food chain. The basis of resistance to these drugs is summarised in Table 1 below.

Fluoroquinolones, notably enrofloxacin, have been shown to be effective against APEC [25], but inevitably, their use leads to the selection for resistance, which tends to persist even in the absence of selection pressure [26]. Quinolones target DNA synthesis in bacteria through targeting of DNA gyrase and topoisomerase V, though resistance in *E. coli* can evolve through alterations to these targets by chromosomal mutations or via active efflux of the drug [27]. Resistance may also be carried on plasmids termed plasmid-mediated quinolone resistance (PMQR), though chromosomal resistance appears to be more frequent [28]. Recent surveys in Korea, Thailand and Australia have shown the prevalence of multi-drug-resistant (MDR) APEC at levels ranging from 70% in Thailand to around 10% in Australia [29,30]. Nearly half of Korean isolates were resistant to enrofloxacin and close to 90% to nalidixic acid, whereas 34% of Thai APEC isolates were resistant to enrofloxacin and around 40% resistant to nalidixic acid. In contrast, only 10% of Australian APEC showed nalidixic acid resistance, indicating greater use of antimicrobials in Asia. As resistance mediated through mutations in gyrase (*gyrA*) has relatively little effect on fitness in *E. coli*, they are more likely to be retained over time [31]. A recent Belgian study found that 40% of APEC isolates showed some resistance to enrofloxacin, with the majority of isolates having gyrase or topoisomerase gene mutations and PMQR making only in a small percentage of resistant isolates [32], whilst a contemporary study in Korea found PMQR in a third of isolates [33]. Quinolone resistance may also be associated with ESBLs and/or colistin resistance, which will be discussed later in the review.

**Table 1:** Examples of antimicrobial resistance genes/mechanisms to clinically important drugs in APEC.

Antibiotic Class	Resistance Mechanisms/Genes	Comments	References
Cephalosporins	AmpC beta-lactamases Including CMY family Extended-spectrum beta-lactamases (ESBLs): CTX-M (CTX-M-1-2-14-15-55) TEM (TEM-52) SHV (SHV-2-12) CTX-M & CMY	plasmid-encoded pAmpC Plasmid and transposon-mediated Both ESBL and AmpC present on plasmid	[34] [29,34,35,36] [34]
Fluoroquinolones	DNA Gyrase (*gyrA*) mutations Topoisomerase V mutations PMQR (plasmid-mediated quinolone resistance)*-qnr* genes	Chromosomally-encoded Plasmid encoded	[27,28,29,30,31,32,33]
Polymixins (colistin)	Mobilised colistin resistance mcr-1	Plasmid encoded	[34,37,38]

Resistance to third and fourth-generation cephalosporins is considered a major public health threat. Enterobacteriaceae resistant to these drugs are commonly found in livestock and domestic animals, representing a clear threat to human health [34]. Resistance to cephalosporins in *E. coli* may result from extended-spectrum beta-lactamases (ESBL) or plasmid-encoded AmpC beta-lactamases (pAmpC), both of which are frequently found in livestock. Both commensal and APEC chicken strains have been shown to carry CTX-M, TEM and SHV ESBL families and pAmpC, mainly of the CMY family [34]. The location of these genes on plasmids and other mobile genetic elements facilitates their spread in production animals where direct transmission, indirect transmission via faeces and entry into the food chain represent a public health risk.

Within APEC, cephalosporin resistance has been recognised as a problem globally. Examples include isolates with *bla*_CTX-M-55_. ESBL in Korea [35] and fluoroquinolone-resistant APEC bearing *bla*_CTX-M-55_ in broiler flocks with recurrent colibacillosis in Japan [36]. Around a quarter of APEC isolates recovered had one of seven different CTX-M genes across 10 different *E. coli* sequence types, indicative of widespread resistance across a range of diverse disease-associated genotypes.

Colistin has been considered a drug of last resort for the treatment of MDR infections. Colistin and other polymixins have a history of high-level veterinary use, both as a therapeutic drug and a growth promoter in Europe and Asia. Although non-therapeutic use was prohibited in the EU from 2011, use in monogastric species remained high in Asia [37]. Perhaps unsurprisingly, pigs and poultry have been shown to be the main reservoirs of key colistin resistance determinants in *E. coli*, including the plasmid-mediated mcr-1 [38], though its specific presence in APEC is not well defined.

## 5. Vaccines for APEC

Vaccination is seen as a key tool as part of wider strategies in reducing the impact of APEC infections in both the layer and broiler sectors, despite the challenges presented by genetic diversity [1]. There are several commercially available vaccines, including the subunit Nobilis INAC and the live attenuated Poulvac vaccines, along with a range of inactivated and especially autologous vaccines. There have been and are a range of experimental approaches to developing APEC vaccines. These include the following:

1. Live attenuated vaccines targeting mutations in either key virulence factors or metabolic pathways.
2. Inactivated vaccines and autogenous vaccines are produced for outbreak strains.
3. Subunit vaccines targeting key virulence factors.
4. Live vector vaccines, including *Lactobacillus* and *Salmonella* vectors expressing APEC antigens.
5. Bacterial ‘ghost’ vaccines and outer membrane vesicles.

### 5.1. Live Attenuated Vaccines

Live vaccines against APEC fall under two main strategies of attenuation. The first is to delete or inactivate individuals or combinations of virulence-associated genes. The second is to mutate key metabolic pathways such as disruption of *aroA* which creates an auxotrophic mutant unable to synthesise aromatic amino acids through disruption of the shikimate pathway, leading to stable attenuation. The development of *aroA* and similar mutations in *Salmonella* has been used to produce a range of experimental and commercial vaccines and vectors. One study showed *galE*, *purA*, and *aroA* mutations in an O78 background delivered via spray-produced serum and mucosal antibodies and gave protection to homologous re-challenge, but not protection against O2 serotype in an aerosol challenge model following low dose infectious bronchitis virus infection [39]. Other metabolic targets include the *crp* and *cya* genes that regulate cyclic AMP in the bacterial cell, singly or in combination as previously used in *Salmonella* vaccines. A double *crp cya* mutant in an O2 strain, but not O78, produced limited protection against pathology [40,41]. A delta *crp* vaccine (Gall N tect CBL) has been developed in Japan, which shows protection against homologous O78 challenge, reduced incidence of colibacillosis, and increased productivity in commercial layer breeders [42,43]. Protection in turkeys from colibacillosis caused by an O2 serotype has also been shown by using mutants in *carAB*, which encodes for carbamoyl phosphatase involved in arginine and pyrimidine biosynthesis [44]. This attenuated mutant reduced mortality, post-mortem lesion scores and detection of the homologous challenge strain in blood following intra-tracheal challenge. The commercially available aroA vaccine itself has been shown to protect against both experimental and field challenges in chickens and turkeys and has been used in combination with autologous vaccines [45,46,47]

Attenuation of virulence factor genes has not been widely explored and is not yet utilised in commercial vaccines, but experimental approaches have identified a mutation in the outer membrane transport gene *tonB*, involved in the transport of siderophores (iron acquisition systems), along with *fur* (a gene involved in iron uptake) as a target [48]. Both single *delta tonB* and double delta *tonB*/delta *furA* strains reduced the numbers of animals with air sac lesions after homologous parent strain aerosols, though neither was fully protective.

### 5.2. Inactivated and Autologous Vaccines

Whilst inactivated vaccines offer a simple solution, the protection offered is limited to homologous challenges within that serotype. Simple bacterins are used in many countries though few other than a liposomal inactivated vaccine delivered by eye drop or spray have been tested with any rigour [49].

Autogenous bacterin vaccines, produced against a specific outbreak strain, can offer protection against an outbreak where current vaccines offer limited protection to that strain. Autogenous vaccines are frequently used in layer and breeder flocks maintained for many months; however, there has been limited study of protection. Homologous but limited heterologous protection to systemic colibacillosis has been demonstrated in an intravenous challenge of mature laying hens [50], but although inactivated autologous vaccines produce antibody responses, they show little protection to egg peritonitis in a reproductive tract challenge model [51]. A combination approach of utilising a commercial vaccine coupled with an autogenous vaccine has shown some promise in reducing infection or pathology with intra-tracheal challenge with O78, O111 and O18 strains [46].

### 5.3. Subunit Vaccines

Vaccines based on individual components, either virulence factors or outer membrane proteins (OMPs), have been explored. Subunit vaccines have good safety records but often struggle to elicit protective responses. One example includes the key virulence factor *iss* (increased serum survival) as a potential target as a vaccine. Recombinant Iss, given intramuscularly, produces a specific immune response and leads to fewer deaths and decreased lesion scores in broiler chickens with a direct air sac challenge with O1, O2 and O78 serotypes [52]. In contrast, the use of the binding domain of FimH, a key adhesin in APEC, as a vaccine elicited an immune response but failed to offer protection to challenge [53]. More recently, reverse vaccinology approaches based on pangenome analysis have identified targets for potential cross-protective protein-based vaccines [54].

A key problem with subunit APEC vaccines is that virulence factors are not associated with all cases of disease in broilers or layers [5], and so protection would not be achieved for all strains. An approach to overcome this is the use of multiple antigenic targets in a single vaccine or to select a commonly expressed antigen not strictly considered a virulence factor. OMPs, or porins, are highly conserved and surface-expressed, so they are considered a potential vaccine target for many Gram-negative bacterial species. OMPs have been used as antigens in outer membrane-delivered vaccines (see below) and together with a siderophore receptor in an SRP vaccine (siderophore receptor porin). The SRP vaccine, primarily intended for use in layers where egg peritonitis is a problem, showed evidence of protection against challenge with O1, O2 and O78 serotypes by a range of challenge routes [55]. A combination of the porins OmpA and OmpT along with another surface protein EtsC and TraT (part of plasmid conjugative apparatus) has been shown to elicit specific IgY and reduce bacterial loads in an acute intra-air sac challenge with an O2 isolate, with serum bactericidal activity to a range of serotypes [56]. The commercially available MSD Nobilis *E. coli* inac is a subunit vaccine containing F11 fimbrial and flagella toxin antigens. As it is intended for broiler breeders, it is designed to provide protection in chicks through maternal antibodies, but there is limited information on its efficacy.

### 5.4. Vector Vaccines

The use of vectors, particularly viral vectors, is well-established in human and veterinary vaccinology. Bacterial vectors, in theory, have advantages in ease of culture, capacity to express multiple antigens and be manipulated for over-expressing of antigens of interest. One of the earliest approaches was to use an established *Salmonella* vaccine strain as a vector expressing O78 LPS, which produced antibody responses and protected against homologous re-challenge [57]. Expression of *E. coli* common pilus antigens in *Salmonella* was used to develop a multivalent vaccine across serotypes [56]. This elicited a strong immune response and a degree of protection for up to three months following the air sac challenge and an attenuated *Salmonella* delivery system for various APEC virulence factors also developed [58].

Expression of the O1 LPS gene cluster in attenuated *Salmonella* Typhimurium leads to specific antibody responses and protection to oral and intramuscular challenge with a homologous strain [59], which was then further developed to produce a bivalent vaccine against O1 and O2 serotypes [60] A *Salmonella* Gallinarum vector vaccine expressing *E. coli* Type 1 fimbrial antigen showed protection to O78 and O161 serotypes along with protection against systemic salmonellosis [61]. Unlike *Salmonella*, members of the genus *Lactobacillus* are both considered part of the healthy intestinal microbiome and key probiotic species, with a history of safety and acceptability to producers and regulators. The concept of using commensal bacteria as vectors is new, but given they have a proven ability to colonise hosts without disease there is merit in the approach. A *Lactobacillus saerimneri* strain isolated from broiler chicks was used to express fimbrial (FimA) and porin (OmpC) antigens from an O78 APEC [62]. Delivered orally, the vaccine candidate produces strong IgY and IgA antibody responses.

### 5.5. Bacterial Ghosts and Outer Membrane Vesicles (OMVs)

Bacterial ghosts are the intact outer membrane structure of Gram-negative bacteria without any cytoplasmic or genetic material contained within. Ghosts are produced by controlled expression of lytic gene *E* of the bacteriophage phiX174, which leads to an intact cell envelope with antigenically intact structures. Ghosts have been proposed as mucosal vaccines and as vaccine vectors, both due to the retention of antigenic structure and due to the presence of multiple pathogen-associated molecular patterns such as lipopolysaccharide (LPS) that act as agonists for pattern recognition receptors. These act to stimulate the innate immune system, producing a considerable adjuvant effect. The advantage of ghosts is that they are non-viable and do not contain genetic material which could be horizontally transferred. However, they lack internal antigens and, as they are non-viable, need repeated doses to elicit immunity. In APEC, ghosts have been proposed as direct vaccines with an O78 ghost offering protection to homologous re-challenge in combination with an infectious bronchitis virus vaccine; similar results have been found with O2 serotypes [63,64]. However, other studies failed to show protection using ghosts [65].

OMVs are small vesicles (up to 300 nm in diameter) formed by budding of the Gram-negative bacterial outer membrane. Like bacterial ghosts, they retain much of the antigenic structure of the bacterial membrane. OMVs may form naturally but can be induced by a range of stressors, including sonication, detergents, heat and antimicrobials. OMVs derived from an APEC O2 strain by ultracentrifugation have been shown to elicit both antibody and Th1 responses and protect against homologous challenge [66]. These OMVs of under 100 nm were taken up well by macrophages and presumably other antigen-presenting cells. A similar approach with O78 isolates also showed good levels of homologous protection [67]. Some protection against the APEC O78 challenge has also been shown following the use of *Salmonella* Typhimurium OMVs as a vaccine [68]

### 5.6. Carbohydrate Conjugate Vaccines

Carbohydrate-based antigens are frequently expressed on the surface of bacterial cells, but their suitability as vaccine targets is somewhat diminished as they are T-cell-independent antigens that fail to elicit responses in neonates. In humans, the use of glycoconjugate bacterial vaccines, where the carbohydrate is conjugated to a protein carrier and turns them into T-cell-dependent antigens, has proved successful in protecting children from a range of meningitis-causing infections. As described above, LPS-based antigens have been incorporated into live vectors [60], but more recently, a synthetic pentasaccharide conjugate based on the repeated subunit of O1 APEC LPS has been developed and tested in vitro, with a view towards the production of a conjugate vaccine [69]. An alternative approach of producing a conjugate based on the siderophore Enterobactin has shown some protection against direct challenge [70] and elicits maternally derived immunity in chicks [71].

## 6. Alternatives to Antimicrobials in APEC Control

### 6.1. Bacteriophage Therapy

The use of lytic bacteriophage to eliminate or reduce levels of APEC has been studied globally [72,73,74]. The extraintestinal nature of APEC, in theory, makes such treatment easier than in the gut, but the variety of APEC genotypes makes targeting the pathogen more difficult. Using a mix or cocktail of phages, and particularly targeting the major genotypes of APEC, is likely to be more successful. Such approaches have shown good efficacy in vitro [75] and in embryo lethality assays when given in ovo [76]. However, treatment of chickens challenged intratracheally with APEC with a cocktail of phage highly effective in vitro failed to offer any protection, although the phage could be recovered from the animals and remained effective in vitro [77]. Furthermore, the emergence of resistance to phage therapy is a barrier to its use and has been demonstrated as occurring rapidly in ST95 O1 APEC isolates treated with phage [78].

### 6.2. Probiotics and Microbiome Modulation

Controls through feed additives such as probiotics are popular in the poultry industry, as they are cheap and easy to administer. As *E. coli* is clearly part of the normal microflora and, indeed, is a key component of the early or pioneer microbiome [79,80], the removal of *E. coli* from the intestinal tract could have unknown ecological consequences [81]. Nevertheless, the use of probiotics or competitive exclusion microflora has been suggested as APEC controls and can be successful in challenge models [82,83], though several other studies are restricted to in vitro inhibition or lack a definition of what APEC is. That said, *E. coli* isolates are among the earliest and most successfully used probiotics; for example, Nissle 1917 and probiotic *E. coli* are likely to display both competitive exclusion and inhibition via bacteriocins [84]. Early colonisation of the gut with beneficial bacterial taxa, including commensal *E. coli*, is likely to lead to increased exclusion of pathogenic APEC strains and enhance immune development and function. Approaches such as in ovo delivery of probiotic mixes or the delivery of microflora, including *E. coli* in the hatchery, are effective against other pathogens, so enhancing the early microbiome rather than specifically targeting APEC may be more effective [83,85,86].

## 7. Conclusions

APEC is a major cause of disease in poultry production, impacting both on meat and egg sectors. Antimicrobial resistance in APEC, particularly ESBL producers, along with fluoroquinolone and colistin resistance, is a major concern for both animal and public health. In the past, antimicrobials were used as much as a prophylactic measure than as a treatment, which is clearly unsustainable. The need for good antimicrobial stewardship has limited the use of antimicrobials both in voluntary schemes such as those in the UK and through legislation in the EU, where flock-level treatment with antibiotics has been banned. As such, alternative measures to better control APEC at the flock level, like vaccination, are needed. The diversity of APEC presents a challenge to vaccine-based control, though our increased understanding of the pathogen and the adoption of multiple approaches to vaccine development offer improved efficacy. The diversity of APEC also poses a challenge in terms of other controls such as probiotics and in the use of bacteriophage needing the selection of appropriate probiotic strains and phage specific for the infecting strain.

With these challenges, APEC is likely to remain arguably the most important bacterial pathogen in terms of animal health within the poultry industry in the next few years. As we better understand genomics and host-pathogen interactions in APEC, better controls may become available to reduce what is a considerable disease burden.

## Data Availability

Not applicable.
